# Identification and Analysis of Hub Genes in Diabetic Cardiomyopathy: Potential Role of Cytochrome P450 1A1 in Mitochondrial Metabolism and STZ-Induced Myocardial Dysfunction

**DOI:** 10.3389/fcvm.2022.835244

**Published:** 2022-03-21

**Authors:** Yinliang Chen, Jinbao Yang, Ying Wang, Weike Shen, Jinlin Liu, Meng Yuan, Xiaoyu Hao, Li Zhong, Rui Guo

**Affiliations:** ^1^College of Life Sciences, Institute of Life Science and Green Development, Hebei University, Baoding, China; ^2^College of Osteopathic Medicine of the Pacific, Western University of Health Sciences, Pomona, CA, United States; ^3^The Key Laboratory of Zoological Systematics and Application, College of Life Sciences, Hebei University, Baoding, China

**Keywords:** hub gene, CYP4501A1, diabetic cardiomyopathy, mitochondrial metabolism, cardiac function

## Abstract

Diabetic cardiomyopathy (DCM) is a primary cause of death in diabetic patients; however, its molecular mechanism is not yet clear, and there is no uniform standard for diagnosis. The aim of this study is to discover the pathogenesis and potential therapeutic targets of DCM through screening and analysis of differentially expressed genes (DEGs) in heart ventricles of DCM, and to testify the role of key hub genes in DCM-induced myocardial dysfunction. Datasets GSE4745 and GSE6880 were downloaded from the GEO database. The difference analysis, visual analysis, cluster analysis and enrichment analysis were performed by using R language, python scripts and bioinformatics software followed by the construction of protein-protein interaction (PPI) network to obtain hub genes. The DCM models were established by streptozocin (STZ) injection to the male mice. The cardiac function and the expressions of hub genes were examined by using echocardiography and real-time quantitative poly-merase chain reaction (RT-qPCR), followed by multiple statistical analyses. Bioinformatic results indicate that mitochondrial dysfunction, disturbed lipid metabolism and decreased collagen synthesis are the main causes of the DCM development. In particular, the hub gene *Cyp1a1* that encodes Cytochrome P450 1A1 (CYP4501A1) enzyme has the highest connectivity in the interaction network, and is associated with mitochondrial homeostasis and energy metabolism. It plays a critical role in the oxidation of endogenous or exogenous substrates. Our RT-qPCR results confirmed that ventricular *Cyp1a1* mRNA level was nearly 12-fold upregulated in DCM model compared to normal control, which was correlated with abnormal cardiac function in diabetic individuals. CYP4501A1 protein expression in mitochondria was also increased in diabetic hearts. However, we found no significant changes in collagen expressions in cardiac ventricles of mice with DCM. This study provided compact data support for understanding the pathogenesis of DCM. CYP4501A1 might be considered as a potential candidate targeting for DCM therapy. Follow-up animal and clinical verifications need to be further explored.

## Introduction

Diabetes is an independent risk factor for cardiovascular diseases (CVDs). The prevalence of heart failure in diabetic patients is as high as 19–26% (1). Persistent high blood glucose and dysregulation in energy metabolism caused by type 1 or type 2 diabetes can result in various cardiovascular complications. Therein, diabetic cardiomyopathy (DCM) is a common clinical complication with a high incidence in diabetic patients in which heart failure can occur at the later stage in the absence of coronary artery disease, hypertension, and valvular heart disease (2). DCM is one of the main causes of death in diabetic patients (3) and characterized by abnormal myocardial structure, myocardial fibrosis, dilated ventricles, diastolic dysfunction, impaired or preserved systolic function, cardiomyocyte hypertrophy and contractile anomalies (2, 4). Despite the increase in the approvement of novel drugs to control diabetes, the prevalence of DCM accompanied with various clinical symptoms has been continuously increasing in the world (5). Moreover, there is no uniform standard for the diagnosis of DCM (6). Thus, more effective treatment or approaches directed at the etiology and pathogenesis underlying DCM are imperative.

The etiology of DCM is complex. Basically, there are two main factors that initiate myocardial damage in diabetics: insulin resistance and hyperglycemia (7). Studies have been shown that the pathogenesis of DCM may implicated with multiple vital events, including metabolic disorders (8), inflammatory response (9), endoplasmic reticulum (ER) stress, oxidative stress (7), mitochondrial damage and dysfunction (10), calcium homeostasis imbalance (11), epigenetics (12), posttranslational modifications and other mechanisms (13). However, researchers have not fully clarified the molecular mechanisms of DCM yet.

It was reported that cytochrome P450 monooxygenases, as one of important sources of reactive oxygen species (ROS), may be regulated in terms of their expressions and activities under diabetic conditions (14), of which, cytochrome P450 1A1 (CYP4501A1) originally observed in the ER, and subsequently found to be localized on the mitochondrial inner membrane (15, 16). Overexpression of *Cyp1a1* attenuated mitochondrial activity and reduced the quality of mitochondrial membranes, while the levels of mitochondrial reactive oxygen species (ROS) were elevated in AC16 cardiomyocytes. These alterations may induce myocardial injuries (15). Additionally, cardiac hypertrophy and impaired vascular were observed in the TCDD (a *Cyp1a1* inducer)-exposed mice accompanied with the upregulation of *Cyp1a1* mRNA expression and ROS accumulation in left ventricles (17). Similarly, knockdown of cytochrome P450 2E1 (*Cyp2e1*), another CYPs family, exhibited the restorative roles in oxidative stress-mediated cardiomyopathy (18). Although the *Cyp1a1* and *Cyp2e1* genes have undesirable effects on the development and survival of cardiomyocytes, their roles in DCM are still unclear.

In contrast to the CYPs which catalyzes the oxidation of exogenous and endogenous substrates (19), collagens were revealed to be participated in maintaining the structure and function of blood vessels. Type I collagens encoded by *Col1a1* and *Col1a2* are essential for maintaining the elasticity of blood vessel walls (20), while type III collagen encoded by *Col3a1* is the main structural component of hollow organs (such as blood vessels, etc.) which plays an pivotal role in sustaining the stretch and tension of tissues (21). However, studies have demonstrated that the myocardial fibrosis derived from the deposition of collagen is correlated with a battery of cardiac diseases, for instance, hypertrophic cardiomyopathy (22), dilated cardiomyopathy (23), and replacement fibrosis following myocardial infarction (24). CTSK has been identified as the most effective cysteine proteinases for the degradation of collagens in mammals, and shown to be upregulated in diabetic cardiomyopathy (4, 25). Hence, the beneficial expression level of collagens for maintaining a healthy cardiovascular system requires further elucidation.

Bioinformatic mining and analysis enables us to screen and identify differential molecular markers from multiple levels of microarray data between healthy and diseased individuals, and it has become an effective research method to explore the potential molecular mechanisms targeting for disease treatment. In this study, we downloaded the gene expression dataset GSE4745 and GSE6880 related to DCM from the Gene Expression Omnibus (GEO) database, and comprehensively proceeded the bioinformatic analyses including the differential analysis, overlapping analysis and clustering analysis to identify the differentially expressed genes (DEGs) between healthy and DCM individuals by using R language and python scripts. The clusterProfiler package of R was then used to perform the GO (Gene Ontology) and KEGG (Kyoto Encyclopedia of Genes and Genomes) enrichment analyses based on their co-expressed differential genes in the corresponding functions and pathways. Furthermore, protein-protein interaction (PPI) network construction was performed by using the combination of STRING online tool and open-source CytoScape software to screen out five hub genes with the highest degree of connection of these DEGs: *Cyp1a1, Cyp2e1, Col1a1, Col3a1, Col1a2*. The flow chart in Supplementary Figure 1 illustrates the gene microarray data mining and analyses by using the combination of R language and bioinformatics online tools. To verify the bioinformatic results, we examined the top five hub genes as well as collagenase cathepsin K (CTSK) levels in heart ventricles by RT-PCR, and detected the cardiac functions in normal and DCM mice. In addition, protein levels of CYP4501A1 and CYP4502E1 expressions in mitochondria were detected. We also performed the correlation analysis between the top-upregulated hub genes (*Cyp1a1* and *Cyp2e1*) and cardiac functional parameters.

## Materials and Methods

### Search Strategy and Information of Microarray Datasets

We used the keyword “diabetic heart” to search the microarray datasets from the GEO database of NCBI (https://www.ncbi.nlm.nih.gov/) (26). Ten pieces of datasets were displayed by restricting the entry type (datasets) and study type (expression profiling by array). We finally selected GSE4745 and GSE6880 as the microarray datasets by further filtration with the disease model (diabetic cardiomyopathy, DCM), sample (heart ventricles), title, summary and the purpose of this study. The GSE4745 dataset was based on the GPL85 platform, [RG_U34A] Affymetrix Rat Genome U34 Array done by Gerber et al. It contains 24 samples of the ventricular gene array data of rats (*Rattus norvegicus*), including 12 controls and 12 diabetics induced by streptozotocin (STZ) injection. In order to sufficiently observe the gene differences between control and diabetic group with typical DCM features, we selected eight samples including four controls and four diabetics on day 42 as the first set of microarray data for the analyses. The cardiac function in diabetic rats on that day showed significant ventricular diastolic and systolic dysfunction. The second dataset GSE6880 was based on the GPL341 platform, [RAE230A] Affymetrix Rat Expression 230A Array, including six samples of the ventricular microarray data of rats (*Rattus norvegicus*), of which, data from three normal hearts and three diabetic heart ventricles were selected as the second dataset for the analyses.

### Data Acquisition and Identification of DEGs

GSE4745 and GSE6880 microarray datasets were downloaded by using the GEOquery package (Version 2.54.1) of R software (https://www.rstudio.com/, Version 3.6.0); and were preprocessed before the screening of DEGs. Briefly, the data with null names or with multiple gene names were not used. As to that the same gene has multiple gene expression results, only the mean values were acquired. These collected data were normalized by utilizing “normalizeBetweenArrays” function, a quantile normalization in R, and the distributions for each set of microarray data were displayed by drawing a boxplot. Subsequently, the normalized microarray data were analyzed by using the limma package of R for DEGs identification. The limma package (Version 3.42.2) requires three matrices for differential analysis: expression matrix, design matrix, and contrast matrix. The design matrix was based on the annotation file of the microarray data and the experimental scheme. The contrast matrix was constructed according to the study groups. We set a threshold at logFC ≥1 (upregulated genes) or logFC ≤ −1 (downregulated genes) to screen out differentially expressed genes. The adjusted *P* < 0.05 was regarded statistically different. The results were saved in CSV file. The DEGs between normal and diabetic groups in datasets GSE4745 and GSE6880 were illustrated by Venn diagrams, from which the overlapped common up- and down-regulated DEGs can be obtained. The distribution and the cluster of the DEGs were visualized by volcano plots and heatmaps, respectively. The heatmap package (version = 1.0.12) was used to numerically normalize the expression levels of DEGs in GSE4745 and GSE6880 datasets in the direction of row normalization.

### GO and KEGG Enrichment Analyses

The GO (Gene Ontology) and KEGG (Kyoto Encyclopedia of Genes and Genomes) enrichment analyses were performed by using the “clusterProfiler” package (Version 3.14.3) of the R language based on the common differentially up- and down-regulated genes of datasets GSE4745 and GSE6880, and an adjusted *P* < 0.05 was considered statistically significant. GO is an international standard classification system for gene functions, including biological process (BP), cellular component (CC) and molecular function (MF) (http://geneontology.org/) (27). KEGG is a database resource that integrates large scale of genomic, chemical and systemic functional information, including but not limited to molecular interaction and metabolic pathways, cellular processes, organismal systems related to human diseases and drug development (https://www.kegg.jp/).

### Gene Set Enrichment Analysis

Gene Set Enrichment Analysis (GSEA) was performed through the GSEA Pre-ranked commands in the GSEA software (http://www.broadinstitute.org/gsea/). The value of log2FC calculated by the limma package was used as the ranking metric. We used the C5 collection which contains gene sets annotated by GO terms, and the C2 pathway gene set collected from KEGG in the analysis. All genes from GSE4745 or GSE6880 were analyzed by GSEA separately, then we analyzed the overlapping pre-ranked enrichment terms which were both in top N (top50 for GO and top20 for KEGG pathway) terms of GSE4745 and GSE6880.

### Construction of PPI Networks

STRING database (https://string-db.org/, Version 11) is a worldwide online resource for the analysis of the interactions among known and predicted proteins. The PPI networks are viable tools to identify key gene modules and core hub genes between patients and healthy individuals. We constructed the PPI networks by importing the common DEGs into the STRING database. The interaction relationships among proteins encoded by these DEGs were then excavated between normal and DCM groups. Subsequently, we applied Cytoscape software to visualize the common DEGs on the basis of the PPI associations, and used the “cytoHubba” plug-in to obtain the hub genes. Top five hub genes were selected according to the ranking order of connectivity degree.

### Animals and Treatment

All studies were performed in accordance with the relevant guidelines and was approved by the Hebei University Animal Care and Use Committee (Approval No. IACUC-2019009XG). DCM model was established by using streptozotocin (STZ) treatment to the mice. Briefly, 9-week-old male C57BL/6N mice were subjected to intraperitoneal injections of STZ (Merck-Sigma-Aldrich®, USA, 100 mg/kg/day) dissolved in 0.1M sterile citrate buffer (adjusting the pH to 4.0 by using 1N NaOH) or vehicle for two consecutive days. Following 4-weeks, fasting blood glucose levels (overnight fasting for 12 hr starting around 9 pm to 9 am) were monitored using a glucometer (CONTOUR®PLUS Blood Glucose Monitoring System, Bayer, Germany). Mice with fasting blood glucose levels >11 mM were deemed diabetic followed by the echocardiographic analysis (28). The female mice were not used because estrogen may have an important role in glucose metabolism after STZ treatment and females are less sensitive to STZ than males (29–31).

### Echocardiographic Analysis

Cardiac geometry and function were evaluated in anesthetized (isoflurane 1.2% administered with a calibrated vaporizer and an inhalant) mice using a two dimensional (2D) guided M-mode echocardiography (Vevo 2100) equipped with a 18–38 MHz linear transducer (Visualsonics, Canada). The heart was imaged in the 2D-mode in the parasternal long-axis view with a depth of 2 cm. The M-mode cursor was positioned perpendicular to interventricular septum and posterior wall of left-ventricle (LV) at the level of papillary muscles from the 2D-mode. Left ventricular diastolic and systolic anterior wall thickness (LVAWD, LVAWS), left ventricular diastolic and systolic posterior wall thickness (LVPWD, LVPWS), ejection fraction (EF), fractional shortening (FS), and LV mass were all measured. Normalized LV mass was calculated as LV mass (mg)/body weight (g).

### Total RNA Extraction, Reverse Transcription, and Quantitative Real-Time PCR

At day 42 (week 6) following STZ injection, total RNA was isolated from ventricles using the TRNzol Universal Reagent (TIANGEN, China). RNAs were quantified using a NanoDrop^TM^ 2000c spectrophotometer (Thermo Fisher Scientific). Synthesis of cDNA and reverse transcription was performed using 1 μg total RNA in a 20-μl system following the instructions of FastKing RT Kit (With gDNase) (TIANGEN, China) and RT SuperMix for qPCR (APE × BIO, USA). *Cyp1a1* and *Rn18s* (a housekeeping gene) primers for qPCR were designed by using the Primer5.0 software. *Cyp2e1* and *Col1a2* primers were referenced from the OriGene's website (https://www.origene.com.cn/). *Col1a1, Col3a1* and *Ctsk* primers were referenced to the previous studies (32–34). The quantitative real-time PCR was performed by using a C1000 Touch Thermal Cycler CFX96^TM^ Real-Time System (BioRad) per the iQ^TM^ SYBR® Green Supermix (CLJC-Bio, China) instructions. Real-time PCR was triplicated for each cDNA sample. The primer sequences are shown in Table 1.

**Table 1:** Primer sequence for RT-qPCR.

**RT-qPCR primers for five hub genes, *Ctsk* and *Rn18S***			**Reference/note**
Primer	Sequence 5′ → 3′	Primer type	
*Cyp2e1*	5′- AGGCTGTCAAGGAGGTGCTACT−3′	Forward	Refer to OriGene's website
*Cyp2e1*	5′- AAAACCTCCGCACGTCCTTCCA−3′	Reverse	
*Cyp1a1*	5′-CCTAACTCTTCCCTGGATGCC-3′	Forward	Designed by Primer 5.0
*Cyp1a1*	5′-TGAGGCTGTCTGTGATGTCCC-3′	Reverse	(NM_009992.4; NM_001136059.2)
*Col1a1*	5′-TGCCTCAGAAGAACTGGTACATCA-3′	Forward	(34)
*Col1a1*	5′-ATCGGTCATGCTCTCTCCAAA-3′	Reverse	
*Col1a2*	5′-TTCTGTGGGTCCTGCTGGGAAA-3′	Forward	Refer to OriGene's website
*Col1a2*	5′-TTGTCACCTCGGATGCCTTGAG-3′	Reverse	
*Col3a1*	5′-TGAAACCCCAGCAAAACAAAA-3′	Forward	(34)
*Col3a1*	5′-TTGGTCACTTGCACTGGTTGAT-3′	Reverse	
*Ctsk*	5′-GGGCCAGGATGAAAGTTGTA-3′	Forward	(33)
*Ctsk*	5′-CACTGCTCTCTTCAGGGCTT-3′	Reverse	
*Rn18s*	5′-GGTGGAGCGATTTGTCTGGTTA-3′	Forward	Designed by Primer5.0
*Rn18s*	5′-CGGACATCTAAGGGCATCACAG-3′	Reverse	(NR_003278.3)

### Mitochondrial Protein Extraction and Western Blot Analysis

The procedures of mitochondrial protein extraction and Western blot was referenced to the previous published methods (35). Briefly, the heart ventricles were homogenized in an ice-cold MSE buffer containing 220 mM mannitol, 2 mM EGTA, 70 mM sucrose, 5 mM Mops (pH 7.4), 0.2% BSA and a protease inhibitor cocktail. The mitochondria pellet was obtained by a gradient centrifugation. The soluble mitochondrial protein fraction was produced by dissolving the mitochondria pellet in the lysis buffer [20 mM Tris/HCl (pH 7.4), 150 mM NaCl, 1 mM EDTA, 1 mM EGTA, 1% Triton, 0.1% SDS and 1% protease inhibitor cocktail] and centrifuged at 10,000 g for 30 min at 4°C (35). A portion (25 μg) of the mitochondrial protein was separated on 12% SDS-polyacrylamide gel, transferred electrophoretically to PVDF membranes and blotted against CYP4501A1 (1:1000, proteintech13241-1-AP), CYP4502E1 (1:4000, proteintech19937-1-AP) and GAPDH (loading control, 1:1000, Cell Signaling Technology) antibodies. After washing with TBST for three times, blots were incubated with horseradish peroxidase (HRP)-conjugated sheep anti-rabbit secondary antibody (1:5000, Cell Signaling Technology) at room temperature with a shaker for 1 h. Antigens were detected by the chemiluminescence method using Bio-Rad imager, and semi-quantitative band analysis was analyzed by Image Lab software (Bio-Rad, Version 5.1, ChemiDoc XRS) (4, 36).

### Statistical Analysis

The bioinformatic statistical analyses were performed in R software (Version 3.6.0). The codes have been uploaded to GitHub at https://github.com/ylchen0622/Paper-data-of-diabetic-cardiomyopathy-.git. The data based on animal experiments were presented as mean ± SEM. Statistical significance (*p* < 0.05) for each variable was estimated by an unpaired *t*-test (two-tailed). Softwares Origin was used for the correlation analysis between the log2-fold change of *Cyp1a1* and *Cyp2e1* mRNA levels and each of the cardiac function variables, as well as the correlation between log2-fold change of *Cyp1a1* and fasting glucose level.

## Results

### Screening and Identification of DEGs Between Healthy and Diabetic Ventricles

**Figure 1:**
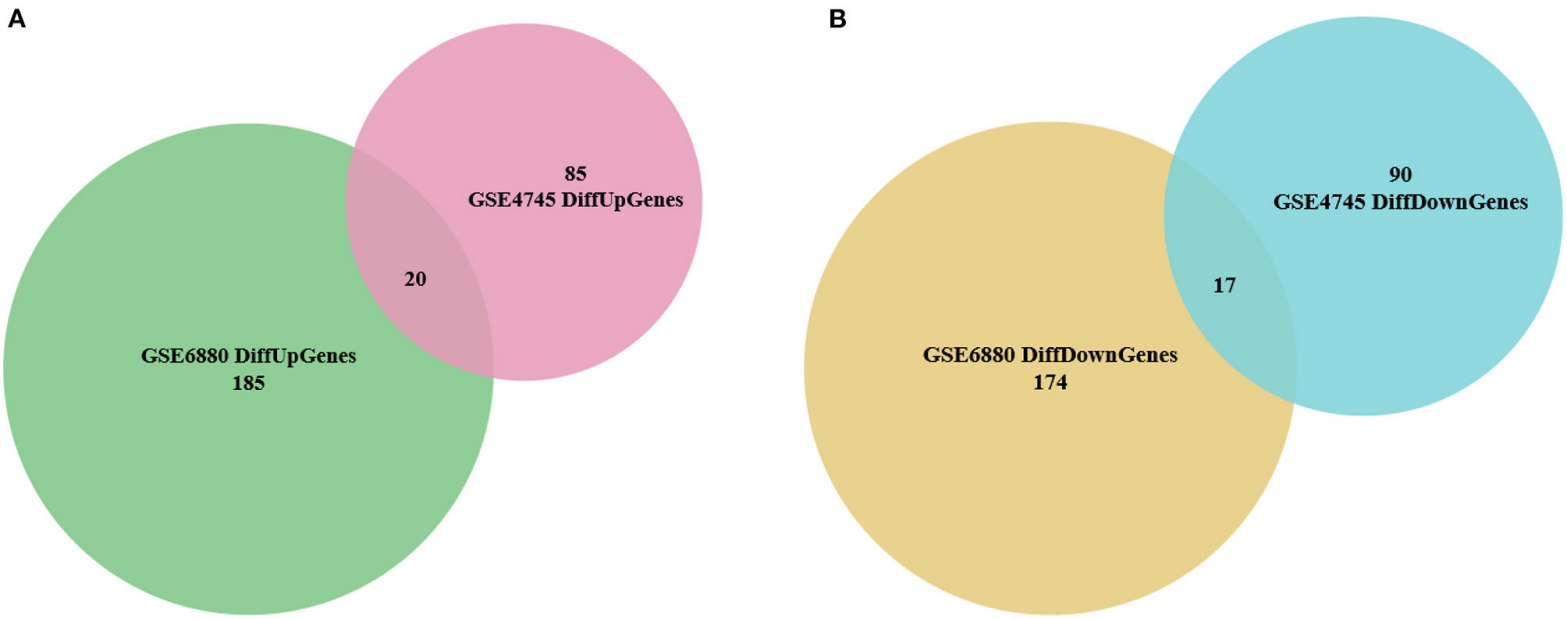
Identification of overlapped differentially expressed genes (DEGs) between normal and diabetic heart samples in datasets GSE4745 and GSE6880. **(A)** Venn diagram of 20 overlapped up-regulated genes in GSE4745 and GSE6880. **(B)** Venn diagram of 17 overlapped down-regulated genes in GSE4745 and GSE6880.

**Figure 2:**
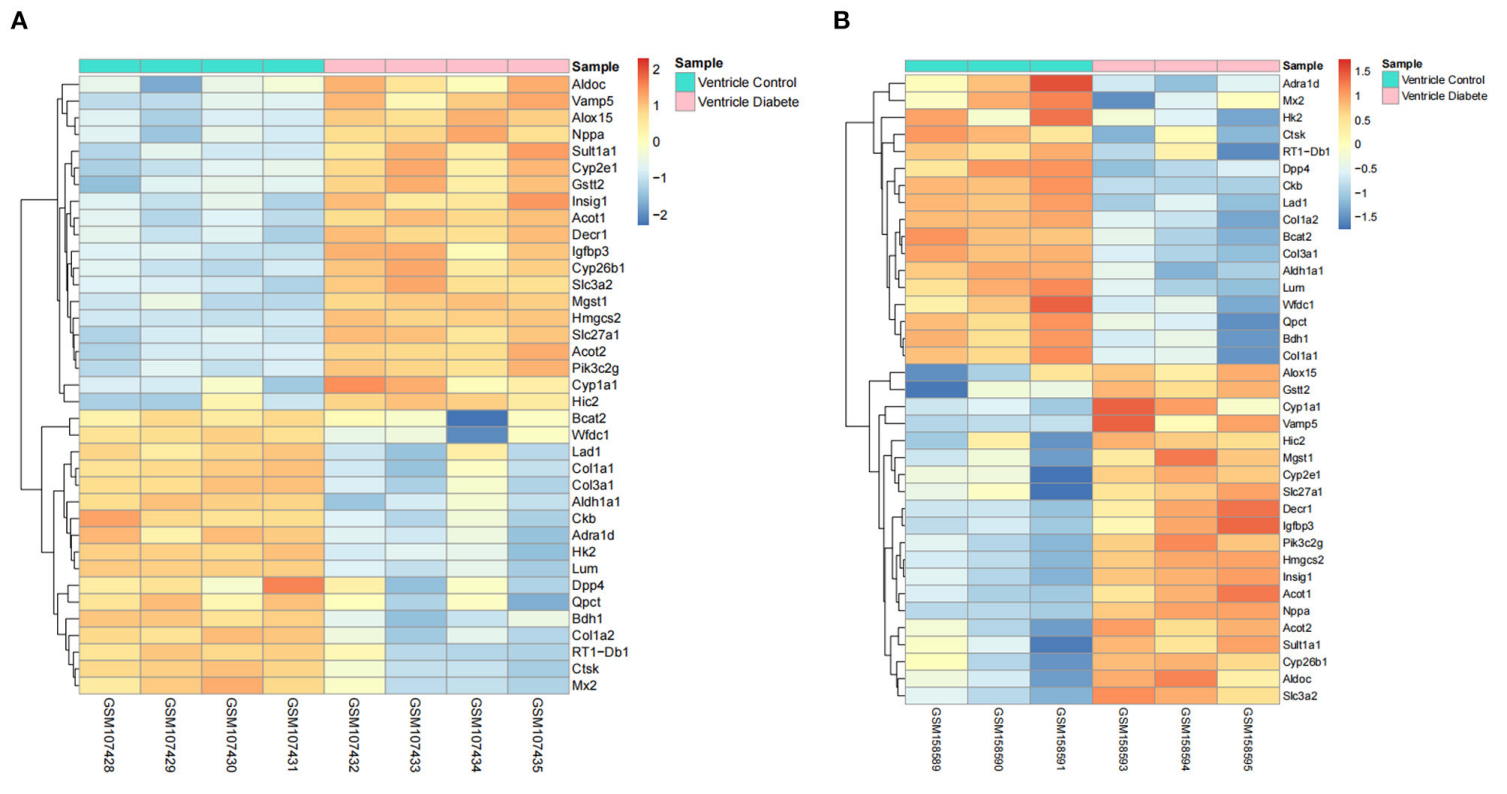
Heatmaps of common DEGs between normal and diabetic heart samples in datasets GSE4745 and GSE6880 (20 up-regulated DEGs and 17 down-regulated DEGs). **(A)** Heatmap of common DEGs in dataset GSE4745. **(B)** Heatmap of common DEGs in dataset GSE6880. The color of the heatmap from blue to red shows the gene expression from low to high. Red indicates upregulated gene expressions. Blue indicates downregulated gene expressions. Pink represents the samples of diabetic group. Turquoise represents the samples of control group.

**Figure 3:**
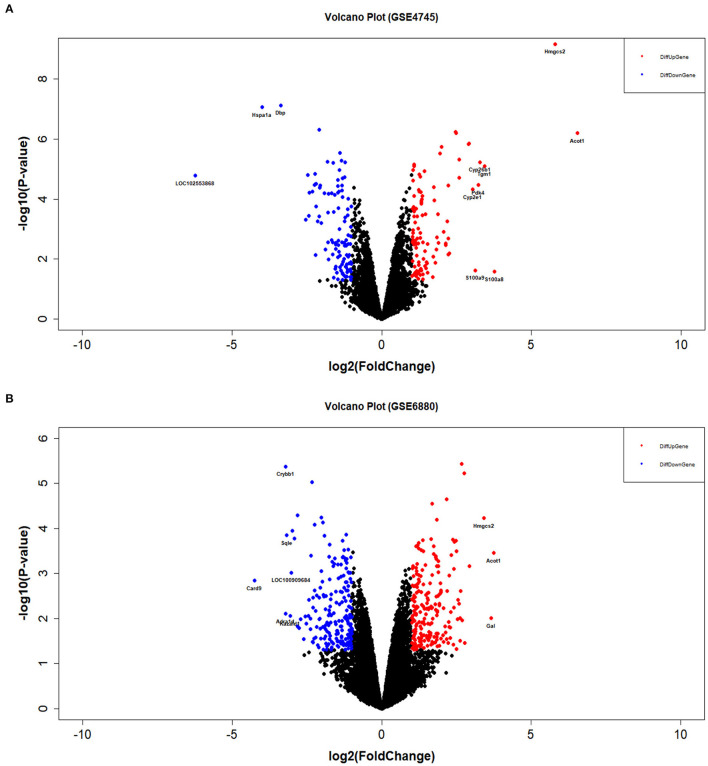
Volcano plots of DEGs between normal and diabetic heart samples in datasets GSE4745 and GSE6880. **(A)** Volcano plot of DEGs in dataset GSE4745. **(B)** Volcano plot of DEGs in dataset GSE6880. The blue dots represent differentially down-regulated genes (logFC ≤ −1 & *P* < 0.05). The red dots represent differentially up-regulated genes (logFC ≥ 1 & *P* < 0.05). FC, fold change.

The distribution profiles of the microarray data in each dataset were matched via quantile normalization and boxplot analysis (Supplementary Figures 2A,B). A total of 212 DEGs (105 up-regulated and 107 down-regulated) in GSE4745, and 396 DEGs in GSE6880 (205 up-regulated and 191 down-regulated) were screened, among which, 37 common DEGs including 20 upregulated genes and 17 downregulated genes were identified in diabetic ventricles compared to normal controls (Figure 1). The names of these genes were listed in Table 2. The DEGs expression of each sample in the two datasets were visualized in the form of a heatmap in Figure 2. In addition, the results of microarray expression matrix in each dataset were displayed in the volcano plots (Figure 3). We screened three significantly down-regulated genes (*LOC102553868, Hspa1a, Dbp*) and eight significantly up-regulated genes (*Acot1, Cyp26b1, Tgm1, Hmgcs2, Pdk4, Cyp2e1, S100a9, S100a8*) in GSE4745 (Figure 3A); six significantly down-regulated genes (*Card9, Adra1d, Crybb1, Sqle, Kazald1, LOC100909684*) and three significantly up-regulated genes (*Hmgcs2, Gal, Acot1*) were screened in GSE6880 (Figure 3B). All comparison data between diabetic group and control (normal) group (diabetic vs control/normal) in datasets GSE4745 and GSE6880 were shown in the Supplementary Excel Files including upregulated and downregulated genes.

**Table 2:** Common differentially expressed genes in GSE4745 and GSE688.

**DEGs**	**Gene terms**
Up-regulated genes (20)	*Insig1 Vamp5 Acot2 Hic2 Cyp1a1 Nppa Igfbp3 Sult1a1 Aldoc Mgst1 Cyp26b1 Cyp2e1 Acot1 Alox15 Slc3a2 Gstt2 Slc27a1 Decr1 Hmgcs2 Pik3c2g*
Down-regulated genes (17)	*Mx2 Bdh1 Ctsk Lum Col3a1 Wfdc1 Bcat2 Aldh1a1 Hk2 Ckb Lad1 Col1a2 Qpct RT1-Db1 Dpp4 Col1a1 Adra1d*

### Enrichment Analysis of Common DEGs

**Figure 4:**
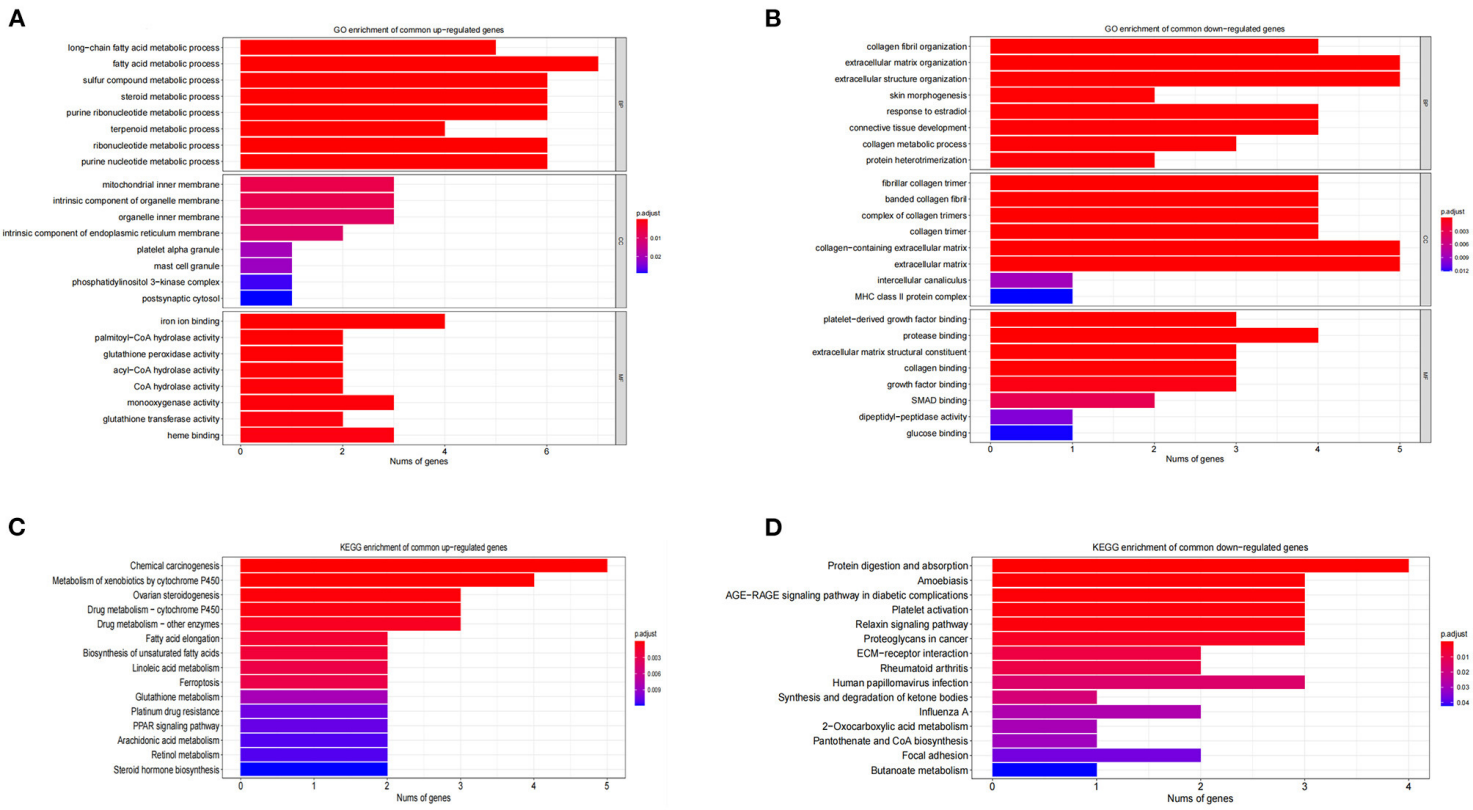
GO and KEGG enrichment analysis of DCM in datasets GSE4745 and GSE6880. **(A)** The enriched GO terms of common differentially up-regulated genes; **(B)** The enriched GO terms of common differentially down-regulated genes; **(C)** KEGG pathway enrichment of common differentially up-regulated genes; **(D)** KEGG pathway enrichment of common differentially down-regulated genes; BP, biological process; CC, cellular component; MF, molecular function.

The GO functional enrichment analysis showed that the common up-regulated DEGs were mainly enriched in fatty acid metabolism during the biological process. At the cellular component level, they were mostly enriched on the inner mitochondrial membranes. In terms of the molecular function, they were chiefly enriched in the iron ions binding and heme binding functions (Figure 4A). Meanwhile, the analysis of KEGG metabolic pathway enrichment showed that these DEGs were mainly enriched in the chemical carcinogenic metabolic process and the metabolism of xenobiotics by cytochrome P450 (Figure 4B). On the contrary, the GO results revealed that common down-regulated DEGs were abundantly present in collagen fiber tissues, extracellular matrix tissues, and extracellular structure tissues. At the cellular component level, they were mostly amassed in collagen fiber trimers and extracellular matrix containing collagens. In respect to the molecular function, they were primarily enriched for the binding of platelet-derived growth factors and the binding of proteases and collagens (Figure 4C). At the KEGG metabolic pathway level, it is mainly enriched in the AGE-RAGE signaling pathway in protein digestion and absorption, and diabetes complications (Figure 4D). Along the same line, GSEA results showed that fatty acid metabolism, xenobiotic metabolism, and ATP synthesis through coupled electron transport chain in mitochondria are the main biological processes enriched in the overlapping terms of GSE4745 and GSE6880. The related pathways (*p* < 0.05) and involved genes, as well as the GSEA overlapping results including biological processes, molecular functions and cellular components were shown in the excel files as our Supplementary Materials.

### PPI Network Analysis and Hub Gene Screening

**Figure 5:**
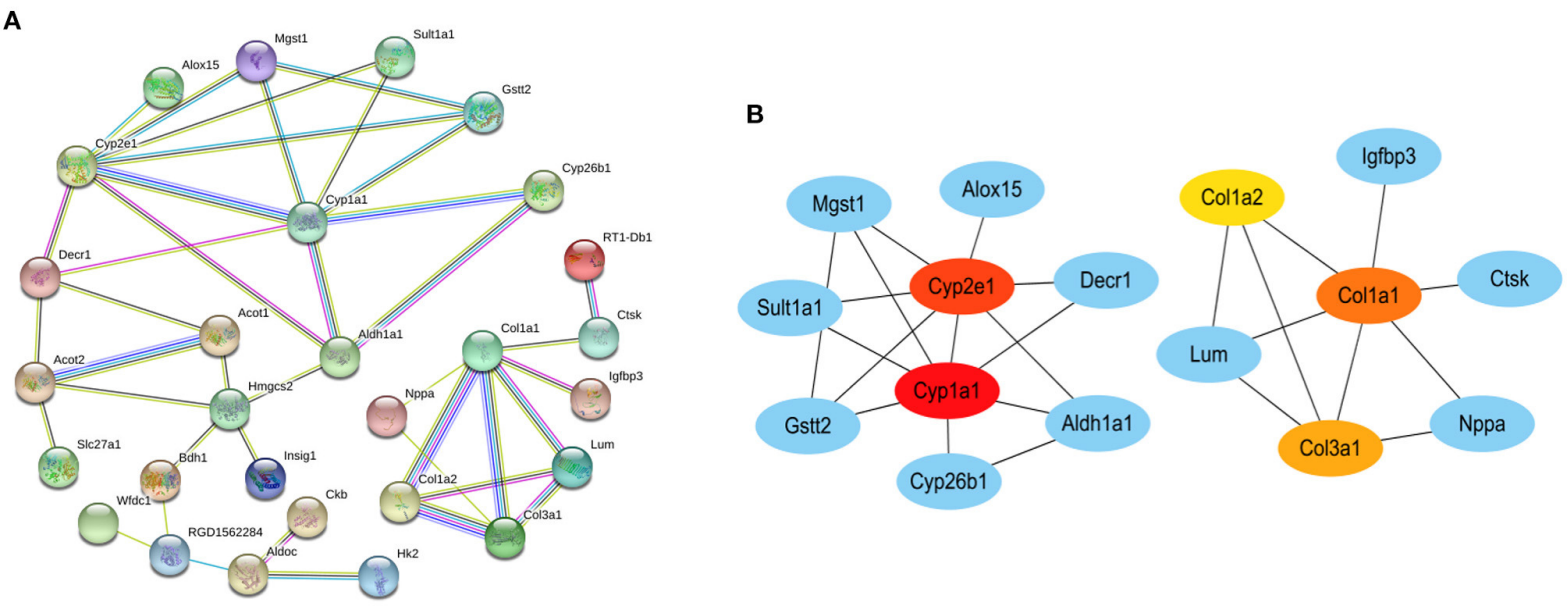
The protein–protein interaction (PPI) networks. **(A)** PPI network of common DEGs in datasets GSE4745 and GSE6880. **(B)** PPI network containing the first five hub genes obtained by cytoscape. The color of the node reflects the degree of connection. The more forward ranking is represented by a redder color. Red represents the highest level. Orange represents the middle level. Yellow represents the lowest level.

We constructed a PPI network by introducing the screened common DEGs into the STRING online analysis software. By removing the unconnected nodes, there were a total of 37 nodes left in the network diagram (PPI enrichment *p* < 1.0^e−16^), suggesting there are multiple biological interactions among these DEGs (Figure 5A). Subsequently, the results of the PPI analysis were imported into the Cytoscape software (Version 3.8.2) to screen out the hub genes. The first five hub genes were selected by using the cytoHubba plug-in function. The sequentially orders are as follows: *Cyp1a1, Cyp2e1, Col1a1, Col3a1, Col1a2* (Figure 5B). These hub genes are closely connected and located at the hub of the PPI network, which are expected to become potential targeted gene candidates for the treatment of DCM.

### General Biometric and Echocardiographic Properties

**Figure 6:**
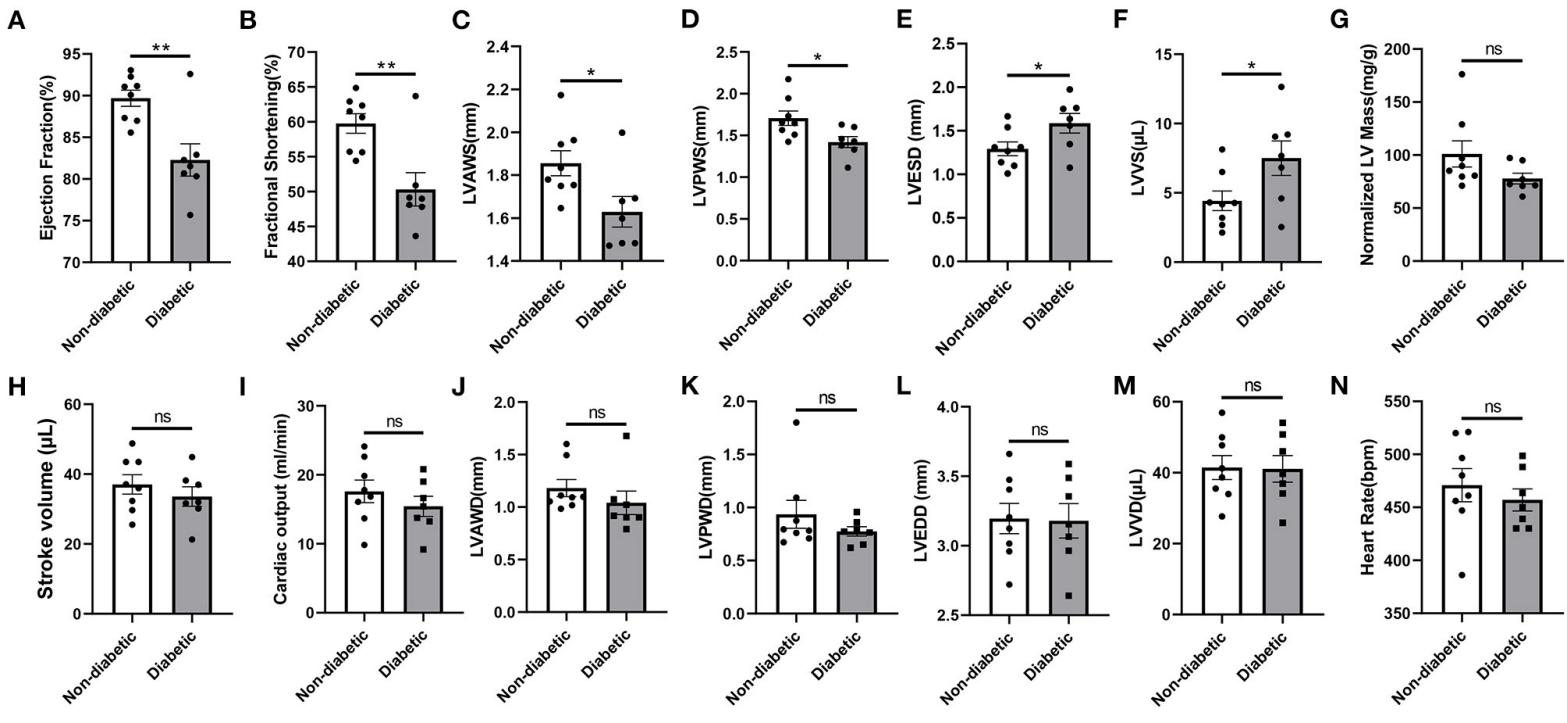
Echocardiographic properties in non-diabetic and STZ-induced diabetic mice. **(A)** Ejection fraction; **(B)** Fractional shortening; **(C)** Left ventricular (LV) anterior wall thickness in systole; **(D)** LV posterior wall thickness in systole; **(E)** LV end-systolic diameter; **(F)** LV volumes in systole; **(G)** LV mass normalized to body weight; **(H)** Stroke volume; **(I)** Cardiac output; **(J)** LV anterior wall thickness in diastole; **(K)** LV anterior posterior wall thickness in diastole; **(L)** LV end-diastolic diameter; **(M)** LV volumes in diastole; **(N)** Heart rate. Mean ± SEM, *n* = 7–8 mice per group. *p < 0.05 and **p < 0.01 vs. Non diabetic control group. ns, no significance.

Following 4 weeks of STZ treatment, the fasting blood glucose level reached to 275.4 mg/dL, which was nearly 2.3 folds higher than in control mice. The water and food consumption were comparably increased (Supplementary Table 1). STZ-induced diabetes caused significant reductions in the ejection fraction (EF), fractional shortening (FS), LV anterior wall thickness in systole (LVAWS) and LV posterior wall thickness in systole (LVPWS) (Figures 6A–D). Meanwhile, the LV end systolic diameter (LVESD) and left ventricular volumes in systole (LVVS) were significantly higher in mice with diabetes (Figures 6E,F). However, the stroke volume (SV), cardiac output (CO), the normalized LV mass, heart rate and related LV parameters in diastole including LVAWD, LVPWD, LVEDD and LVVD were not markedly altered following STZ injections (Figures 6G–N). The representative M mode and B mode images were shown in Supplementary Figure 3. Following 6 weeks of STZ treatment, the liver mass and size were significantly increased, while the spleen mass and size were dramatically reduced compared to the control. STZ failed to affect the body weight, heart weight and kidney weight themselves. However, the kidney to body weight was shown to be increased (Supplementary Table 2).

### Levels of Cardiac Hub Genes and *Ctsk* in the Non-diabetic and Diabetic Mice

**Figure 7:**
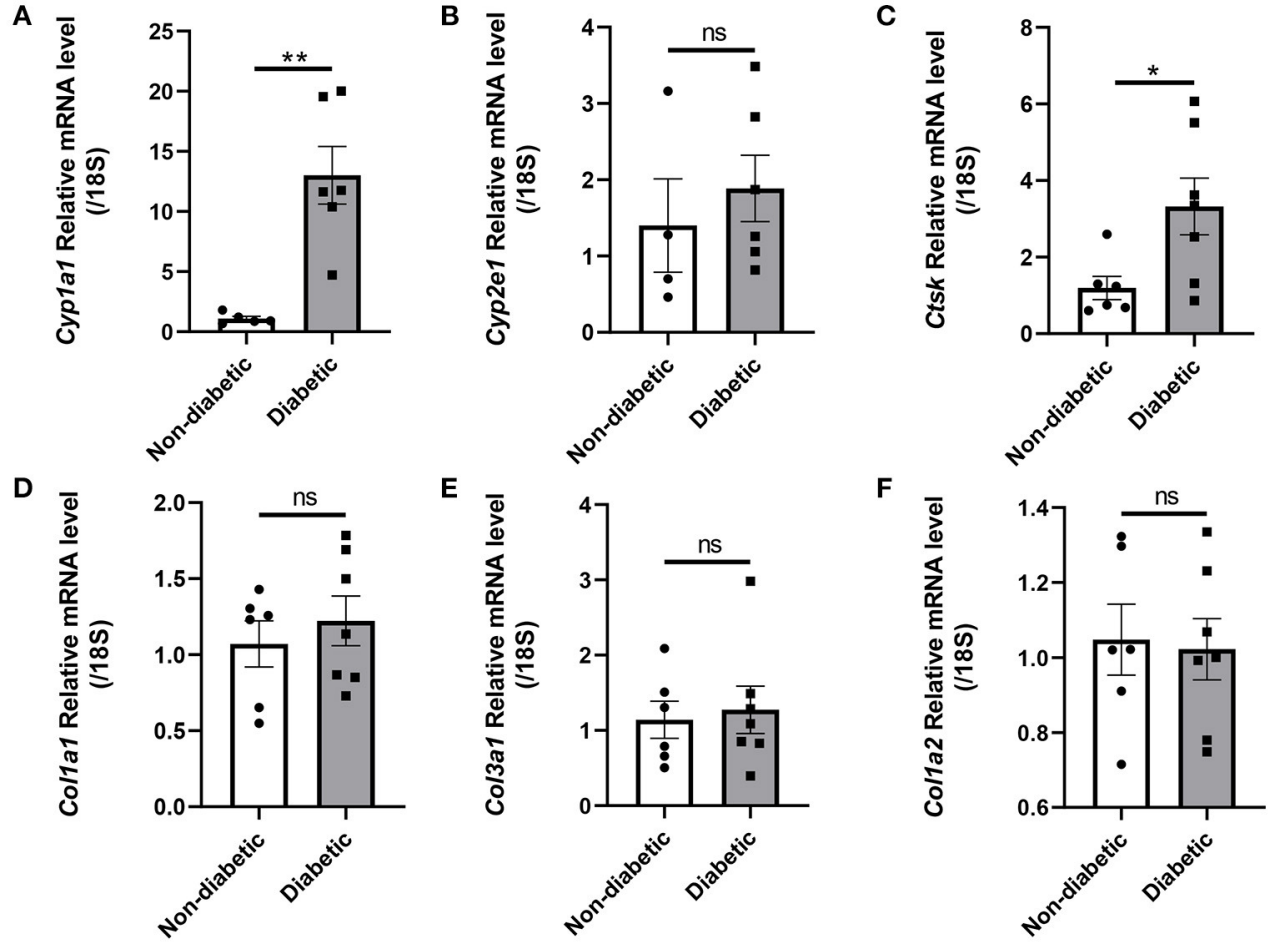
Hub genes and *Ctsk* expression in the hearts of non-diabetic and STZ-induced diabetic mice. **(A)** *Cyp1a1* mRNA fold change; **(B)** *Cyp2e1* mRNA fold change; **(C)** *Ctsk* mRNA fold change; **(D)** *Col1a1* mRNA fold change; **(E)** *Col3a1* mRNA fold change; **(F)** *Col1a2* mRNA fold change. Mean ± SEM, *n* = 4–7 mice per group. *p < 0.05 and **p < 0.01 vs. Non diabetic control group. ns, no significance.

Levels of hub genes *including Cyp1a1, Cyp2e1, Col1a1, Col3a1* and *Col1a2*, as well as *Ctsk* were measured in heart ventricles of control and STZ induced diabetic mice using quantitative real-time PCR. *Cyp1a1* and *Ctsk* mRNA expressions were elevated by about 12-fold change and 2-fold change, respectively. However, *Cyp2e1, Col1a1, Col3a1 and Col1a2* had no obvious difference in diabetic mice than non-diabetic subjects (Figures 7A–F).

### Correlations Between *Cyp1a1*/*Cyp2e1* and Cardiac Functions in Non-diabetic and Diabetic Mice

**Figure 8:**
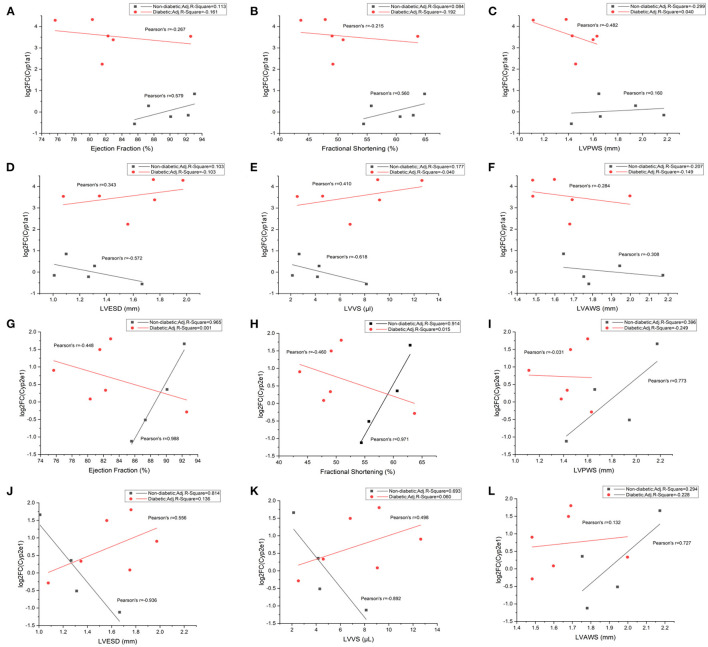
Correlations between *Cyp1a1*/*Cyp2e1* mRNA levels and cardiac functional parameters in non-diabetic and STZ-induced diabetic mice. **(A)** Correlations between log2-fold change (log2FC) of *Cyp1a1* and ejection fraction; **(B)** Correlations between log2FC of *Cyp1a1* and fractional shortening; **(C)** Correlations between log2FC of *Cyp1a1* and LVPWS; **(D)** Correlations between log2FC of *Cyp1a1* and LVESD; **(E)** Correlations between log2FC of *Cyp1a1* and LVVS; **(F)** Correlations between log2FC of *Cyp1a1* and LVAWS; **(G)** Correlations between log2-fold change (log2FC) of *Cyp2e1* and ejection fraction; **(H)** Correlations between log2FC of *Cyp2e1* and fractional shortening; **(I)** Correlations between log2FC of *Cyp2e1* and LVPWS; **(J)** Correlations between log2FC of *Cyp2e1* and LVESD; **(K)** Correlations between log2FC of *Cyp2e1* and LVVS; **(L)** Correlations between log2FC of *Cyp2e1* and LVAWS. *n* = 4–6 mice per group.

Our analyses showed that *Cyp1a1* had a positive correlation with ejection fraction, fractional shortening, and LVPWS in non-diabetic mice. On the contrary, a negative correlation was illustrated between them in diabetic mice (Figures 8A–C). At the same time, it revealed a negative correlation between *Cyp1a1* and LVESD or LVVS in non-diabetic mice but a positive correlation in diabetic mice (Figures 8D,E). Additionally, we observed negative correlations between *Cyp1a1* and LVAWs in both diabetic and non-diabetic groups (Figure 8F). Similarly, there was a positive correlation between *Cyp2e1* and EF, FS or LVPWS in non-diabetic mice, while a negative correlation between them in diabetic mice (Figures 8G–I). *Cyp2e1* was negatively correlated with LVESD and LVVS in non-diabetic mice but positively correlated with them in diabetic mice (Figures 8J,K). However, we observed positive correlations in both groups between *Cyp2e1* and LVAWs (Figure 8L). In addition, correlations between *Cyp1a1* mRNA levels and fasting glucose in non-diabetic and diabetic mice were also shown in Supplementary Figure 4.

### Protein Levels of Mitochondrial CYP4501A1 and CYP4502E1

Protein levels of mitochondrial CYP4501A1 and CYP4502E1 were measured in heart ventricles of non-diabetic and STZ induced diabetic mice by using immunoblot analysis. CYP4501A1 expression in mitochondria were significantly increased in diabetic heart compared with control. While there was no significant change of mitochondrial CYP4502E1 expression between two groups although there was an increased trend induced by STZ (Supplementary Figure 5).

## Discussion

Diabetes mellitus is reaching epidemic proportions globally and is bringing significant socioeconomic burden to countries and regions. DCM occurs because of uncontrolled hyperglycemia and dysregulated lipid metabolism associated with diabetes. However, currently, there is no efficient strategy for the DCM diagnosis and treatment before the onset of heart failure. This is partially due to the asymptomatic manifestation in diabetic patients for the first several years, as well as its complicated molecular mechanisms. The salient findings from the present study support that *Cyp1a1*, a significantly upregulated DEG, has the highest connectivity in the PPI interaction network among the top five hub genes (*Cyp1a1, Cyp2e1, Col1a1, Col3a1, Col1a2*) to affect the cardiac function in STZ-induced diabetic cardiomyopathy. Although similar work has been reported, and the different hub genes were identified based on distinct datasets, the methodologies and searching strategies for the dataset selection were not the same (37). In this study, we strictly focused on “diabetic cardiomyopathy (DCM)” and “heart ventricles” as our main searching restriction conditions to specify the disease stages and the tissue samples. We observed the gene expression through the microarray matrix and statistically analyzed the expression differences of tens of thousands of genes at the same time. The statistical power generated by this method is higher than that obtained by other statistical methods, which greatly reduces the occurrence of errors (38). Through the GO enrichment analysis, we found the common down-regulated DEGs were significantly enriched in extracellular matrix remodeling by proteases, and the AGE-RAGE signaling pathway in diabetic complications. On the contrary, the common up-regulated DEGs were dominantly located in the inner mitochondrial membrane, enriched in fatty acid metabolism, and they mainly function in the iron and heme binding. Particularly, the KEGG enrichment analysis suggested that the majority of the up-regulated DEGs were significantly enriched at the level of cytochrome P450-mediated xenobiotic metabolism, ferroptosis, fatty acid elongation and biogenesis, which may be associated with DCM development. These were also supported by the GSEA results. From these findings, we predicted that CYP450 may play a potential role in mitochondria-mediated fatty acid metabolism and associated with ferroptosis.

Cytochrome P4501A1 (CYP4501A1) and cytochrome P4502E1 (CYP4502E1) belong to the cytochrome P450 family and play important roles in the generation of ROS. Overexpression of *Cyp1a1* or *Cyp2e1* can produce excessive ROS which in turn promotes fatty acid peroxidation and inhibits the activity of mitochondrial respiratory chain, leading to cardiomyocyte injury (15, 39, 40). As a NADPH dependent enzyme, CYP4501A1 catalyzes electron transport systems in mitochondria, regulates the metabolism of xenobiotics, and mediates cell lipid peroxidation (41–43). Mitochondria are rich in mammalian cardiomyocytes. Perturbations of mitochondrial metabolism may markedly influence cardiomyopathies. Our data revealed that diabetes dramatically induced dilated cardiomyopathy and cardiac systolic dysfunction characterized by diminished ejection fraction, decreased wall thickness in systole, as well as increased end systolic diameter and volume. These myocardial anomalies were accompanied with a significant elevation in *Cyp1a1* mRNA expression in heart ventricles compared to normal control, indicating an adverse impact of CYP4501A1 overload on the myocardium, which is consistent with our bioinformatic results. Interestingly, our results also revealed an increased level of CYP4501A1 in mitochondria of heart ventricles after STZ administration, suggesting that STZ-induced diabetes can cause CYP4501A1 overload in mitochondria, which may aggravate mitochondrial metabolic burden and thus promote oxidative stress (44–46). Additionally, we found two forms of CYP4501A1 expressed in mitochondria of STZ-induced diabetic heart, the higher molecular weight may indicate a posttranslational modification of CYP4501A1 (16, 47), although further experiments and verification are still needed. These results were also supported by the scenario that overexpression of CYP4501A1 in cardiomyocytes can increase mitochondrial ROS and has a detrimental effect on mitochondrial quality control and cardiomyocyte injury (15). Moreover, data from this study demonstrated that *Cyp1a1* expression is negatively correlated with ejection fraction, fractional shortening and posterior wall thickness in diabetic mice, whereas they are positively correlated in non-diabetic mice. Along the same line, *Cyp1a1* expression is positively correlated with LV end systolic diameter and volume in diabetic groups but has negative correlations with LVESD and LVVS in non-diabetic control group. In addition, the data distribution has no intersection between non-diabetic and diabetic individuals. These findings indicate that CYP4501A1 has a vital impact on diabetes-induced left ventricular systolic dysfunction, and the posterior wall thickness is more susceptible to be affected than the anterior wall in DCM. Consistently, there was a higher expression of *Cyp2e1* in diabetic ventricles, although it was not significant, the same trend of correlations with those cardiac parameters was found in both groups, suggesting CYP4501A1 may exhibit more important role than CYP2E1 in regulating the cardiac function in diabetics. Moreover, through the analysis of the common DEGs in the GSE4745 and GSE6880 datasets, CYP4501A1 has the most extensive connectivity throughout the network, and thus has dramatic impact on the DCM development over other genes or proteins. In accordance with our bioinformatic results, persistence of high glucose in diabetic individuals stimulates *Cyp1a1* that may directly or indirectly modulate *Cyp2e1, Decr1, Mgst1, Gstt2, Sult1a1, Cyp26b1, Aldh1a1* or *Alox15*, and subsequently damage mitochondria, disrupt lipid and glucose metabolism, resulting in the accumulation of harmful products, the induction of ferroptosis, and eventually myocardial damage.

It was demonstrated that CYP4501A1 is targeted to mitochondria through specifically cleaving the corresponding signal peptide sequences by proteases in the cytoplasmic matrix such as metalloprotease (47, 48). Contrary to the datasets GSE4745 and GSE6880 from the original articles, we detected a significant upregulation of cathepsin K in diabetic hearts, which is consistent with our previous study (4). High glucose may trigger the release of cathepsin K from lysosomes to the cytosol, which in turn stimulate other biological activities such as apoptotic cell death (49). Although we have not yet to further discovered the regulatory role of cathepsin K in targeting of CYP4501A1 to mitochondria, this is worth to be further explored in the future. In addition, based on different studies, the changes in the levels of collagens of diabetic heart is controversial. Unlike the reduced expression of *Col1a1, Col3a1* and *Col1a2* in GSE4745 and GSE6880 datasets (38, 50), we failed to find significant alterations of *Col1a1, Col3a1* and *Col1a2* levels between non-diabetic and diabetic hearts, indicating less effect of these collagens on the myocardial defects. The upregulation of cathepsin K, the most matrix-degrading cysteine proteinases, may be another cause to affect the collagen expressions in the diabetic heart (51, 52). On the contrary, some researchers also reported slight or significant upregulations of collagen I and III in diabetic heart, contributing to the cardiac fibrosis (53–55).

## Summary and Conclusion

In conclusion, among the five hub genes we identified from GEO datasets, *Cyp1a1* presented the highest connection in the PPI network. The significant upregulation of CYP4501A1, as well as the impairment of mitochondrial function and fatty acid metabolism may exhibit pivotal role in the occurrence of DCM in the murine hearts. The results and data analyses from this study not only provide the support for understanding of the pathogenesis of DCM, but also research directions and targets for further basic or clinical exploration. The follow-up work in our lab is to testify the effect of *Cyp1a1* deletion on the progress of DCM, and to investigate the potential molecular mechanisms using transgenic mice and cell lines.

## Strengths and Limitations

The strengths of our methodology include: the utilization of the overlapping results from two datasets for the gene screening; using multiple enrichment analyses including GO, KEGG and GSEA; narrowing down the genes of interest to those that contribute significantly to the gene network; the exploration was made for the verification between different species. However, although bioinformatics and the analysis of the DEGs in gene expression microarrays is a powerful approach to study the connection between genes and diseases, the datasets themselves may also have certain shortage and deviations. Moreover, the aim of the bioinformatic analyses is only to provide data support and clues for the subsequent experimental studies. There may be a certain deviation between the results of bioinformatics analyses and the actual experimental results. Other factors can be also involved to affect the results such as sample size and animal species. In addition, we cannot exclude the role of other genes and pathways we screened out in the development of DCM. Some potential regulators such as non-coding RNA or microRNA may also play important role in the regulation of certain gene expressions or be participated in some molecular signaling pathways to affect the DCM pathogenesis.

## Data Availability Statement

The datasets presented in this study can be found in online repositories. The names of the repository/repositories and accession number(s) can be found in the article/Supplementary Material.

## Ethics Statement

The animal study was reviewed and approved by Hebei University Animal Care and Use Committee Approval No. is IACUC-2021XG032.

## Author Contributions

YC and RG: conceptualization, methodology, design of the research, writing, and original draft preparation. YC and WS: bioinformatic data collection and analysis. JY, YW, JL, and XH: experimental data collection. JY, YW, MY, JL, and RG: experimental data analysis. YC, JY, WS, and RG: software validation and result interpretation. YC, JY, and RG: figures preparation. RG and LZ: reviewing and revising and editing. All authors approved the final version of the manuscript.

## Funding

This work was supported by the National Natural Science Foundation of China (#31900534 and #32171181, RG), the Natural Science Foundation of Hebei Province (#C2019201349, RG), the Hundred Talents Funding Program of Hebei Province (#E2019050010, RG), and the Advanced Talents Incubation Program of the Hebei University (#801260201282, RG).

## Conflict of Interest

The authors declare that the research was conducted in the absence of any commercial or financial relationships that could be construed as a potential conflict of interest.

## Publisher's Note

All claims expressed in this article are solely those of the authors and do not necessarily represent those of their affiliated organizations, or those of the publisher, the editors and the reviewers. Any product that may be evaluated in this article, or claim that may be made by its manufacturer, is not guaranteed or endorsed by the publisher.
