# Supplementary material for: Identification and Analysis of Hub Genes in Diabetic Cardiomyopathy: Potential Role of Cytochrome P450 1A1 in Mitochondrial Metabolism and STZ-Induced Myocardial Dysfunction
Source: Front Cardiovasc Med. 2022 Mar 21;9:835244. doi: 10.3389/fcvm.2022.835244 (PMC8977650; doi:10.3389/fcvm.2022.835244)
Supplement: Supplementary file 3 [file Data_Sheet_1.pdf]

Supplementary Material

Supplementary Figures and Tables

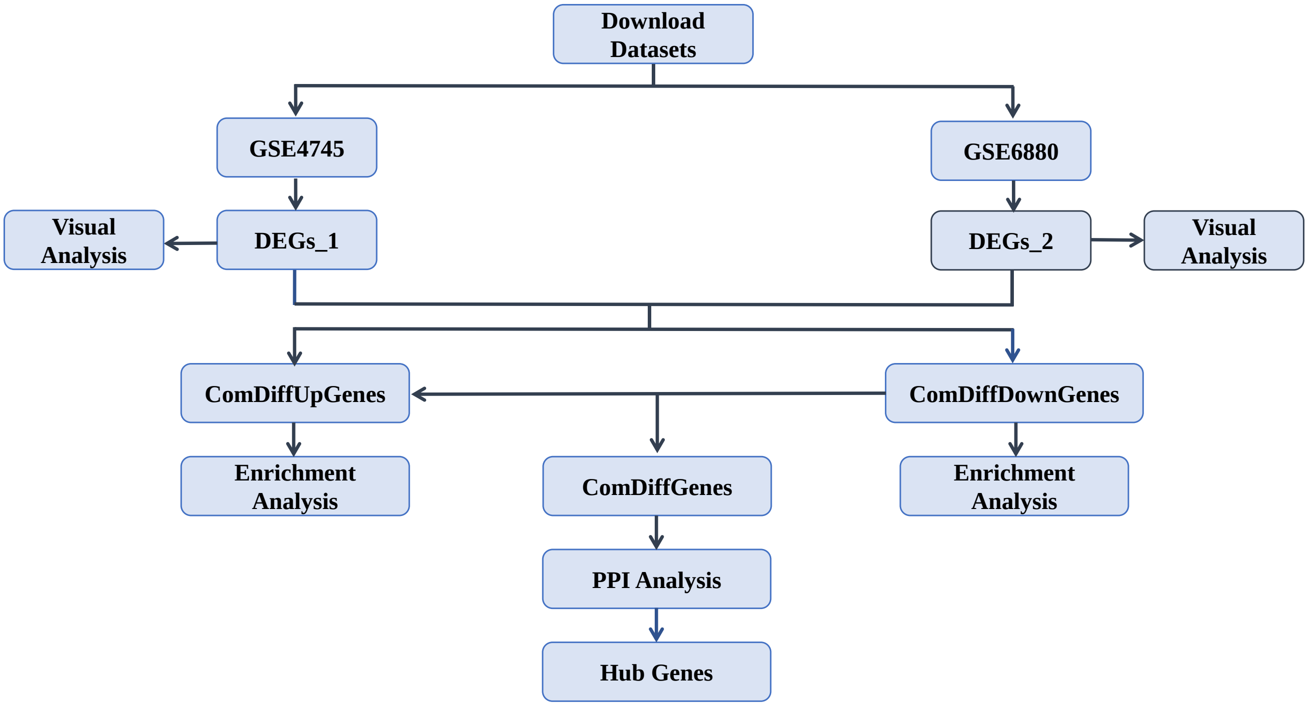

Supplementary Figure 1. Flow chart of screening and analysis of differentially expressed genes in diabetic cardiomyopathy based on bioinformatics. ComDiffUpGenes: Common differentially up-regulated genes; ComDiffDownGenes: Common differentially down-regulated genes; ComDiffGenes: Common differentially expressed genes.

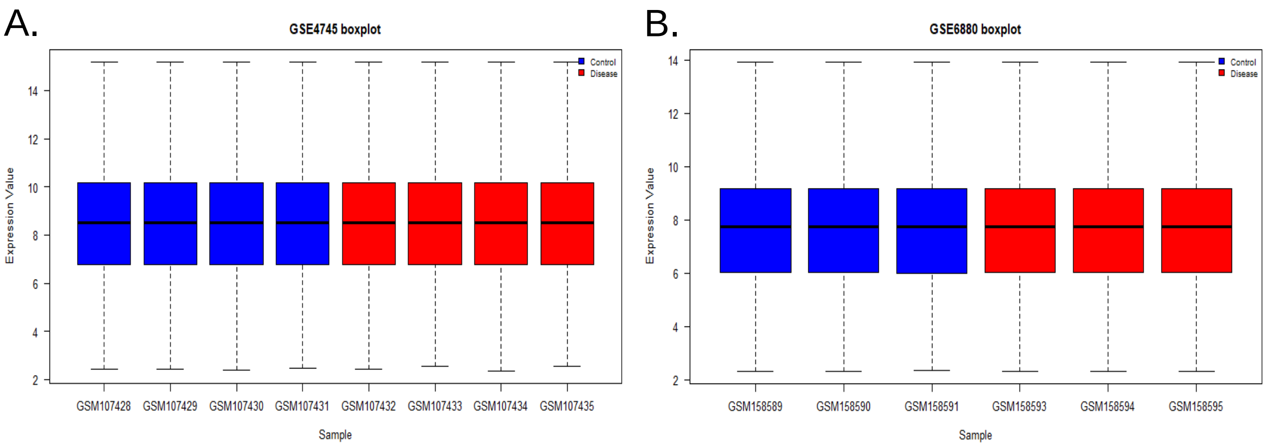

Supplementary Figure 2. Matched distribution profiles of the microarray data. (A) Matched distribution of microarray data in GSE4745. (B) Matched distribution of microarray data in GSE6880.

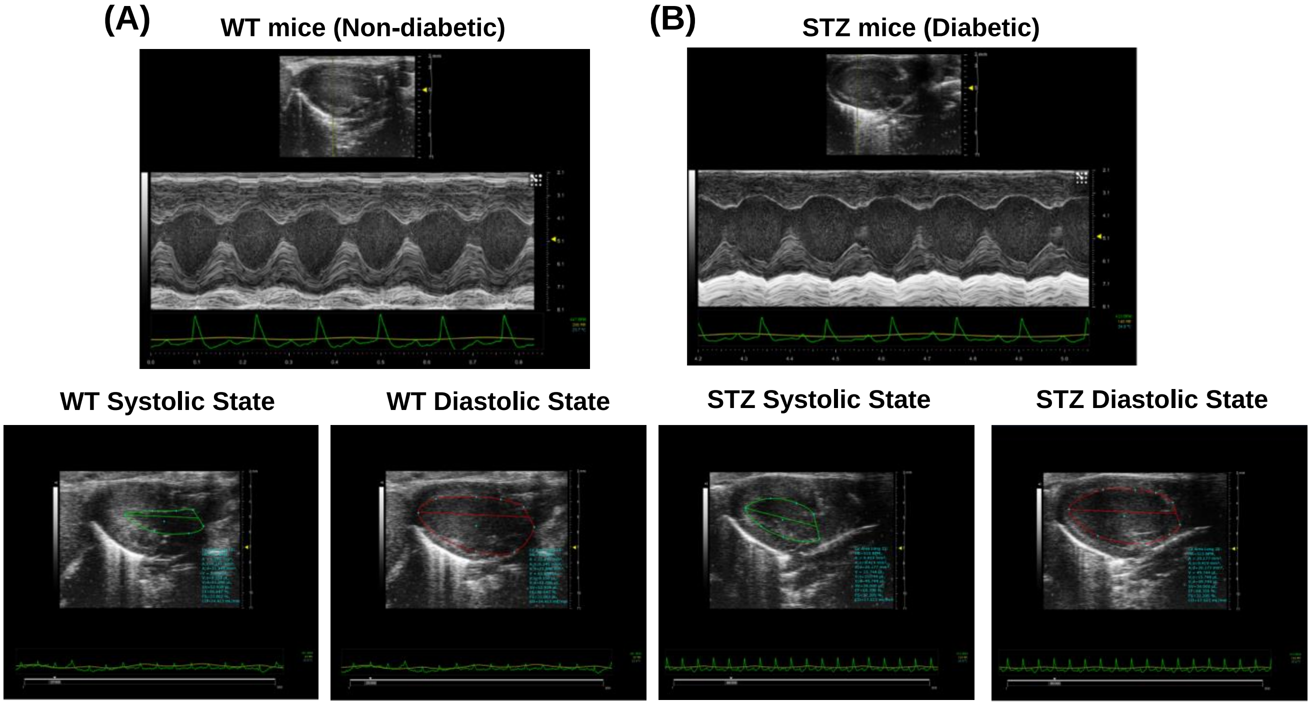

Supplementary Figure 3. Representative echocardiographic images of M mode and B mode under systolic and diastolic states in WT non-diabetic and STZ-induced diabetic mice. (A) Representative echocardiographic images in WT non-diabetic mice. (B) Representative echocardiographic images in STZ-induced diabetic mice.

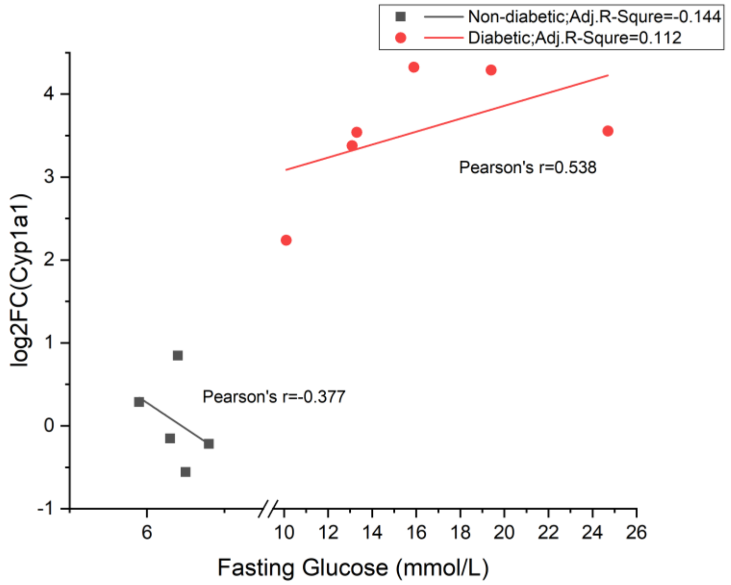

Supplementary Figure 4. Correlations between *Cyp1a1* mRNA levels and fasting glucose in non-diabetic and diabetic mice. Correlations between log2-fold change (log2FC) of *Cyp1a1* and fasting glucose. n= 5–6 mice per group.

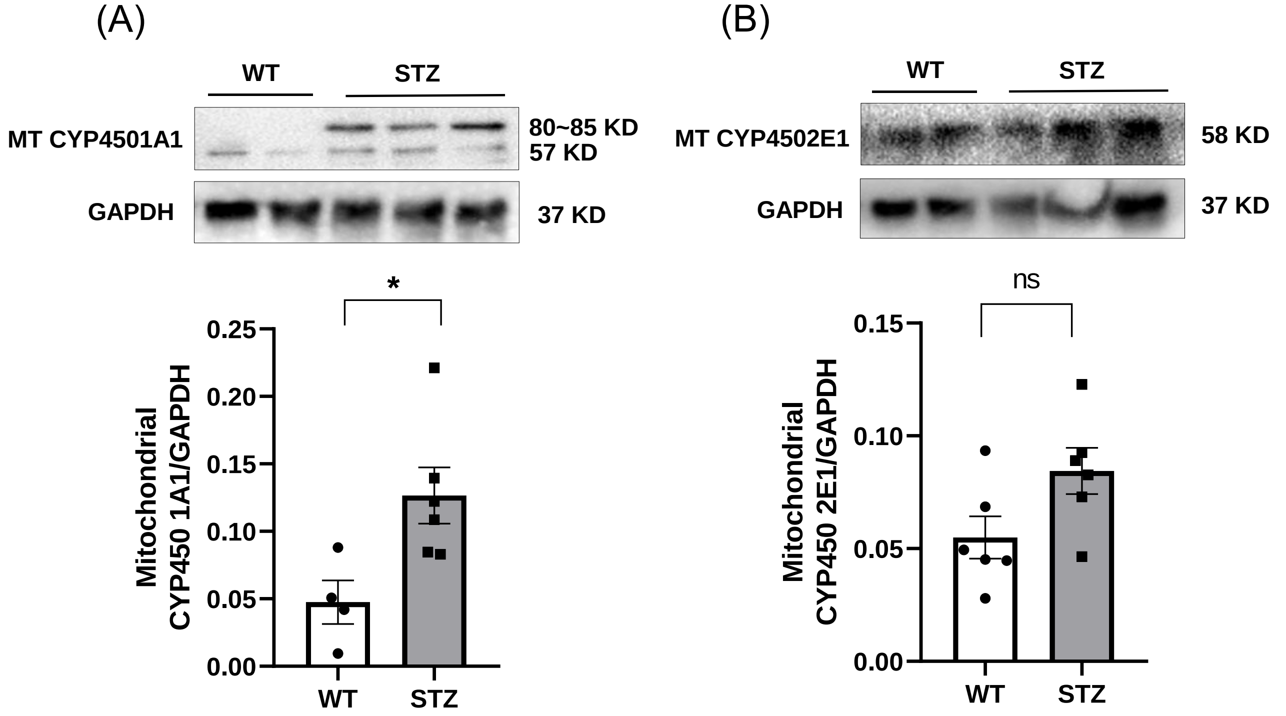

Supplementary Figure 5. Mitochondrial CYP4501A1 and CYP4502E1 expression in the hearts of wild type (WT) non-diabetic and STZ-induced diabetic mice. (A) Mitochondrial CYP4501A1 expression normalized by GAPDH; (B) Mitochondrial CYP4502E1 expression normalized by GAPDH; Mean ± SEM, n= 4–6 mice per group. *$p<0.05$ vs. WT group.

Supplementary Table 1. Diabetic profiles of C57 mice following 4-weeks of STZ-treatment

Parameter	WT	WT-STZ
Fasting blood glucose (mg/dL)	118±3.2	275±36***
Water consumption (mL/d)	3.5±0.17	11.5±2.6*
Food consumption (g/d)	3.3±0.20	4.6±0.47*

Mean ± SEM, n=7 to 8 mice per group. *$p<0.05$ versus WT-group; ***$p<0.001$ versus WT-group.

Supplementary Table 2. General features of C57 mice following 6-weeks of STZ-treatment

Parameter	WT	WT-STZ
Body weight (g)	24.7±0.3	23.1±0.8
Heart weight (mg)	159±10	136±8
Liver weight (g)	1.14±0.04	1.31±0.04*
Kidney weight (g)	0.33±0.01	0.35±0.01
Spleen weight (mg)	68±4	51±3**
Heart/ body weight (mg/g)	6.42±0.35	5.87±0.27
Liver/ body weight (mg/g)	46.3±1.4	56.6±0.5****
Kidney/body weight (mg/g)	13.5±0.4	15.4±0.4**
Spleen/body weight (mg/g)	2.73±0.14	2.22±0.07**

Mean ± SEM, n=7 to 8 mice per group. *$p<0.05$ versus WT-group; **$p<0.01$ versus WT-group; ****$p<0.0001$ versus WT-group.
